# Phenotypic and Functional Properties of Porcine Dedifferentiated Fat Cells during the Long-Term Culture *In Vitro*

**DOI:** 10.1155/2015/673651

**Published:** 2015-05-18

**Authors:** Xuewu Peng, Tongxing Song, Xiaoming Hu, Yuanfei Zhou, Hongkui Wei, Jian Peng, Siwen Jiang

**Affiliations:** ^1^Department of Animal Nutrition and Feed Science, College of Animal Science and Technology, Huazhong Agriculture University, Wuhan 430070, China; ^2^The Cooperative Innovation Center for Sustainable Pig Production, Wuhan 430070, China; ^3^Key Laboratory of Swine Genetics and Breeding of Agricultural Ministry and Key Laboratory of Agricultural Animal Genetics, Breeding and Reproduction of Ministry of Education, College of Animal Science and Technology, Huazhong Agriculture University, Wuhan 430070, China

## Abstract

It has been proved that terminally differentiated mature adipocytes possess abilities to dedifferentiate into fibroblast-like progeny cells with self-renewal and multiple differentiation, termed dedifferentiated fat (DFAT) cells. However, the biological properties of DFAT cells during long-term culture *in vitro* have not been elucidated. Here, we obtained fibroblast-like morphology of porcine DFAT cells by ceiling culture. During the dedifferentiation process, round mature adipocytes with single large lipid droplets changed into spindle-shaped cells accompanied by the adipogenic markers *PPARγ*, *aP2*, *LPL*, and *Adiponectin* significant downregulation. Flow cytometric analysis showed that porcine DFAT cells displayed similar cell-surface antigen profile to mesenchymal stem cells (MSCs). Furthermore, different passages of porcine DFAT cells during long-term culture *in vitro* retained high levels of cell viabilities (>97%), efficient proliferative capacity including population doubling time ranged from 20 h to 22 h and population doubling reached 47.40 ± 1.64 by 58 days of culture. In addition, porcine DFAT cells maintained the multiple differentiation capabilities into adipocytes, osteoblasts, and skeletal myocytes and displayed normal chromosomal karyotypes for prolonged passaging. Therefore, porcine DFAT cells may be a novel model of stem cells for studying the functions of gene in the different biological events.

## 1. Introduction

Mesenchymal stem cells (MSCs) are multipotent cells derived from the stromal fraction of many adult tissues including bone marrow [1], adipose tissue [2], skeletal muscle [3], peripheral blood [4], and umbilical cord [5]. MSCs possess the capacity of self-renewal and multipotency to differentiate into adipocytes, osteoblasts, and chondrocytes [6]. Compared with bone marrow derived mesenchymal stem cells (BMSCs), adipose-derived stem cells (ASCs) isolated from adipose tissue have been an attractive source of MSCs due to the easy accessibility with little donor site injury [7]. However, the heterogeneity and insufficient cell numbers of ASCs limit the clinical applications.

Mature adipocytes constitute more than 90% of adipose tissue volume and are the most abundant cell type in adipose tissue [8]. Recent studies have reported that terminally differentiated mature adipocytes possess ability to undergo dedifferentiation and form proliferative, multipotent, and fibroblast-like progeny cells by ceiling culture, named dedifferentiated fat (DFAT) cells [9, 10]. DFAT cells exhibit similar properties to BMSCs and may be a novel adult stem cell source for tissue engineering and cell therapy [11] because of the more abundant cell numbers and the high purity as compared to ASCs [12]. However, the functional characteristics of DFAT cells during long-term culture* in vitro* remain elusive. Pigs are generally considered as large animal model which are similar to human in genetic and physiological characteristics and could be an ideal model for biomedicine.

In the present study, we attempt to harvest porcine DFAT cells by ceiling culture of mature adipocytes isolated from adipose tissue. Then, we identify biological characteristics of porcine DFAT cells including morphology, viability, immunophenotype, differentiation potential, and chromosomal karyotypes during the long-term culture* in vitro*, aiming to further define the properties of porcine DFAT cells by prolonged passaging.

## 2. Materials and Methods

### 2.1. Isolation, Purification, and Culture of Porcine Mature Adipocytes

All animal experiments were approved by the Animal Care and Use Committee of Huazhong Agricultural University, China. Mature adipocytes were isolated from five-day-old male landrace piglet with a slight modification described previously [9]. Briefly, subcutaneous adipose tissue was obtained in sterile condition and washed with phosphate-buffered saline (PBS). Then, the adipose tissue was minced into small pieces and digested in 0.1% (w/v) collagenase type I (Sigma, USA) at 37°C for 1 h with gentle agitation. After filtration and centrifugation at 300 g for 10 min, the floating mature adipocytes in the top layer were collected. After the wash with Dulbecco's modified Eagle's medium (DMEM, Invitrogen, USA) for three times, cells (5 × 10^4^) were plated in 12.5 cm^2^ culture flasks (Jet, Canada) completely filled with DMEM supplemented with 20% fetal bovine serum (FBS, Invitrogen, USA) and were incubated at 37°C in 5% CO_2_. Culture flasks containing mature adipocytes were inverted and the floating adipocytes attached to the top inner ceiling surface.

To obtain highly purified mature adipocytes, cells were purified with differential plating until there were no other cells to eliminate contamination [13]. After 7 days of ceiling culture, the culture flasks were inverted so that the cells were on the bottom and the medium was changed every 3 days until the cells reached confluence.

Primary DFAT cells were digested with 0.25% (w/v) trypsin-EDTA (Invitrogen) and long-term cultured in growth medium of low-glucose DMEM supplemented with 15% FBS, 10 ng/mL basic fibroblast growth factor (bFGF) (Peprotech, USA), and 1% penicillin/streptomycin (Invitrogen, USA).

### 2.2. Flow Cytometric Analysis

Porcine DFAT cells at passage 4 (P4) were detected by flow cytometric for cell surface antigen. The cells were digested with 0.25% trypsin-EDTA and incubated with primary antibody CD29 (552369, BD, USA), CD31 (MCA1746PET, AbD Serotec, USA), CD34 (GB12013, China), CD44 (ab19622, Abcam, USA), and CD90 (562245, BD, USA) for 30 min at 4°C and further they were incubated for 30 min at 4°C with FITC or CY3 conjugated secondary antibody. Cell fluorescence was detected by FACSCalibur instrument (Becton Dickinson, USA). The data were analyzed using CellQuest Software (Becton Dickinson, USA).

### 2.3. Cell Viability Assay

The cell viability was assessed by trypan blue staining. The stain of the porcine DFAT cells was performed according to the manufacturer's instructions of Countess Automated (Invitrogen, USA).

### 2.4. Growth Kinetics Analysis

Growth kinetics of porcine DFAT cells were analyzed by detection of cumulative population doubling (CPD) and population doubling time (PDT). Porcine DFAT cells at P1 were plated at 1 × 10^4^/cm^2^ in growth medium until 70%–80% confluence and the cells were digested and replated at 1 × 10^4^/cm^2^ in growth medium. The calculation of population doubling was performed as the formula: PDs = log_2_ (number of harvested cells/number of seeded cells) [11]. Cumulative population doubling (CPD) was calculated by the total numbers of PDs in the previous passages. PDT was calculated by the formula: PDT = *t*/PDs, where *t* is the culture time during this passage [14].

### 2.5. RNA Extraction, Reverse Transcription, and Real-Time PCR

Total RNA was extracted using TRIzol reagent (Invitrogen, USA) according to the manufacturer's instructions. Total RNA (1 *μ*g) was performed by reverse transcription (RT) using reverse transcription kit (Toyobo, Japan). Real-time PCR was performed with iTaq Universal SYBR Green Supermix (Bio-Rad, USA) using the CFX96 Detection System (Bio-Rad, USA). The relative expression levels of* PPARγ*,* aP2*,* LPL*,* Adiponectin*, and* Runx2* mRNA were normalized to *β*-actin. The primers are as follows: *β*-actin forward: 5′-CCAGGTCATCACCATCGG-3′, reverse: 5′-CCGTGTTGGCGTAGAGGT-3′;* PPARγ* forward: 5′-AGAGTATGCCAAGAACATCC-3′, reverse: 5′-AGGTCGCTGTCATCTAATTC-3′;* aP2* forward: 5′-AAGTCAAGAGCACCATAACC-3′, reverse: 5′-GATACATTCCACCACCAACT-3′;* LPL* forward: 5′-CGACTCTCTGTTGAATGAAG-3′, reverse: 5′-TTGGCTCTGACCTTATTGATC-3′;* Adiponectin* forward: 5′-TTGAAGGATGTGAAGGTCAG-3′, reverse: 5′-CAATGTTGTGGTAGAGAAGG-3′;* Runx2* forward: 5′-CAGACCAGCAGCACTCCATA-3′, reverse: 5′-AACGCCATCGTTCTGGTTAG-3′;* MyoG* forward: 5′-CCAACCAGCGGCTGCCTAAAG-3′, reverse: 5′-ATTGTGGGCGTCTGTAGGGTCA-3′.

### 2.6. Adipogenic, Osteogenic, and Myogenic Differentiation

For adipogenic differentiation, DFAT cells at 80%–90% confluence were treated with high-glucose DMEM containing 10% FBS, 1 *μ*M dexamethasone, 500 *μ*M isobutylmethylxanthine (IBMX), 10 *μ*g/mL insulin, and 200 *μ*M indomethacin (all reagents were from Sigma, USA) for 12 days. The lipid droplets were confirmed by Oil Red O stain and extracted total RNA for RT-PCR analysis.

For osteogenic differentiation, DFAT cells at 50%–60% confluence were induced with high-glucose DMEM containing 10% FBS, 0.1 *μ*M dexamethasone, 10 mM *β*-glycerophosphate, and 50 mM ascorbic acid (all reagents were from Sigma, USA) for 21 days and induction medium was replaced every 3 days. The mineralized deposits were visualized by Alizarin Red stain and extracted total RNA for RT-PCR analysis.

For myogenic differentiation, DFAT cells at 70%–80% confluence were induced with low-glucose DMEM containing 200 ng/mL Galectin-1 (Prospec, USA) protein for 21 days, and medium was changed every 3 days. Myogenic markers Desmin and MyHC were assessed by immunofluorescence staining.

### 2.7. Immunofluorescence Analysis

Myogenic differentiation of DFAT cells was detected by immunofluorescence staining. The induced cells were fixed with 4% paraformaldehyde for 10 min and treated with 0.1% Triton X-100. Then, cells were blocked with 5% bovine serum albumin (BSA, Sigma, USA) and incubated with primary antibodies Desmin (1 : 100, sc-14026, Santa Cruz, USA), and MyHC (1 : 100, ab11083, Abcam, USA) overnight at 4°C. Thereafter, TRITC and FITC conjugated secondary antibodies were incubated at room temperature for 1 h. The cell nucleus was stained with DAPI.

### 2.8. Karyotype Analysis

Chromosomal karyotyping of early, middle, and late passages of DFAT cells was performed as previously reported [15]. At least 20 metaphases were acquired by Leica microscope.

### 2.9. Statistical Analysis

The results are presented as means ± standard deviations. All experiments were repeated a minimum of three times. The statistical significance was assessed by one way analysis of variance (ANOVA) along with Duncan multiple range test. *p* values < 0.05 were considered significant.

## 3. Results

### 3.1. Morphological and Gene Expression Changes during the Ceiling Culture of Porcine Mature Adipocytes

**Figure 1:**
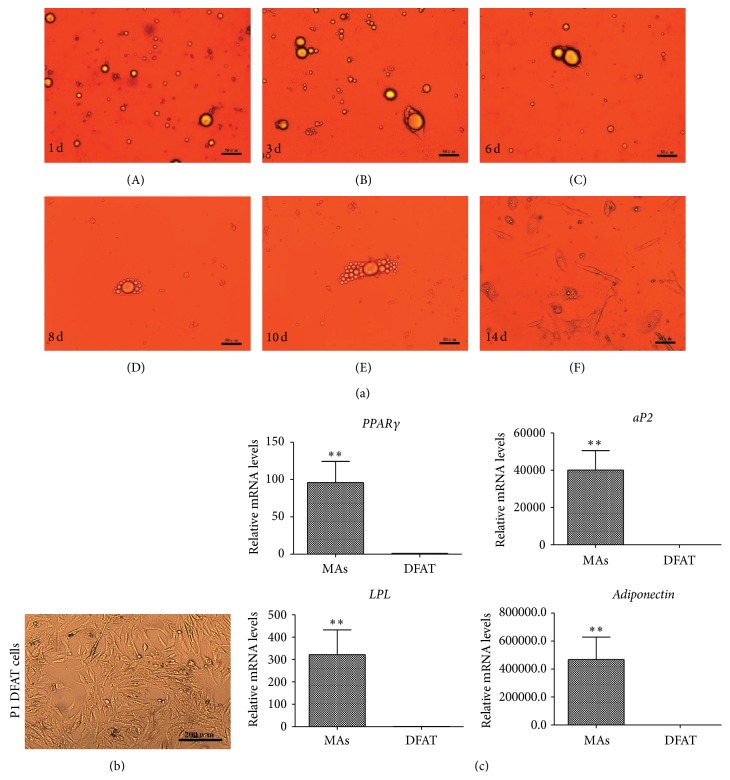
Morphological and gene expression changes during the dedifferentiation from porcine mature adipocytes to DFAT cells by ceiling culture. (a) Morphological changes of the dedifferentiation process. During the ceiling culture, mature adipocytes contained large single lipid droplets attached to the flasks firmly (A) at days 1-2 and the cells became oval shape at day 3 (B). Then, the lipid droplets were released and extruded out the cells at days 6–10 (C–E). Finally, the cells changed into fibroblast-like DFAT cells (F), bar, 50 *μ*m. (b) Primary DFAT cells at P1 after trypsinization, bar, 200 *μ*m. (c) The gene expression of adipogenic markers between mature adipocytes (MAs) and DFAT cells. The mRNA relative levels of qPCR* PPARγ*,* aP2*,* LPL*, and* Adiponectin* were detected by qPCR. Values are the mean ± SD of triplicate dishes.  ^∗∗^ *p* < 0.01.

Porcine mature adipocytes were isolated and purified with collagenase digestion and differential plating method. During the ceiling culture, the mature adipocytes containing single large lipid droplets (Figure 1(a)-A) attached to the top surface of the culture flasks in 1-2 days and changed morphology in following about 2 weeks (Figure 1(a)-A–F). After mature adipocytes firmly attached to the flasks, the shape became to elongated and spread “tentacles” at day 3. Then, the single large lipid droplets in the cytoplasm gradually were released and extruded out the cells on days 6–14, leading to the following: the cell changed into fibroblast-like morphology and did not contain lipid droplets (Figure 1(a)-F). The culture flasks were inverted, the medium was changed at days 7–10, and the cells proliferated and reached confluence. After trypsinization, P1 DFAT cells uniformly exhibited fibroblast-like morphology and we refer to these cells as DFAT cells in this paper (Figure 1(b)).

Next, we detected the gene expression changes of dedifferentiation of mature adipocytes (MAs) during ceiling culture by using real-time RT-PCR. Adipogenic related genes including* PPARγ*,* aP2*,* LPL*, and* Adiponectin* were highly expressed in mature adipocytes, whereas these genes were almost not expressed in DFAT cells (Figure 1(c)). These results suggest that DFAT cells lose the functional characteristics of mature adipocytes and are consistent with previous reports [9, 11].

### 3.2. Cell-Surface Antigen Expression Profile of DFAT Cells

**Figure 2:**
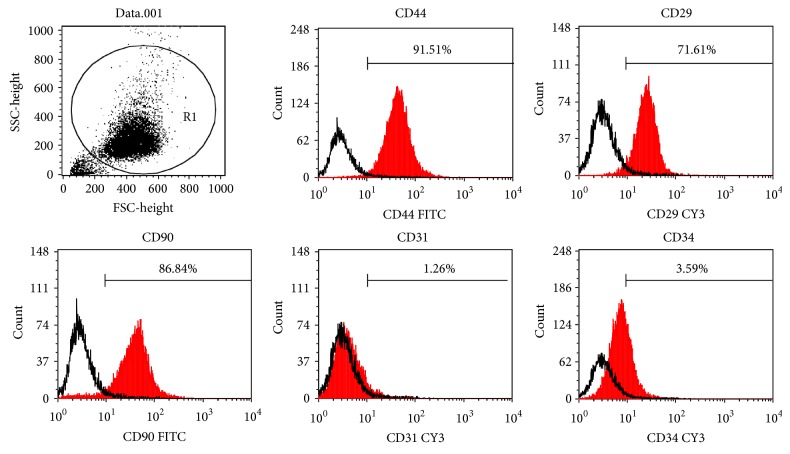
Expression of cell surface antigens on porcine DFAT cells. Expressions of CD44, CD29, CD90, CD31, and CD34 were detected by FITC or CY3 conjugated antibody of P4 DFAT cells.

The cell-surface antigen of porcine DFAT cells at P4 was examined by flow cytometric analysis. DFAT cells were positive for mesenchymal surface antigen including CD44 (91.51%), CD29 (71.61%), and CD90 (86.84%), which are typically expressed in MSCs. The expression levels of endothelial cells marker CD31 (1.26%) and hematopoietic cells marker CD34 (3.59%) were low in porcine DFAT cells (Figure 2). This expression profile was similar to BMSCs and consistent with that previously reported [9, 11].

### 3.3. Cell Morphology and Viability of Porcine DFAT Cells during Long-Term Culture

**Figure 3:**
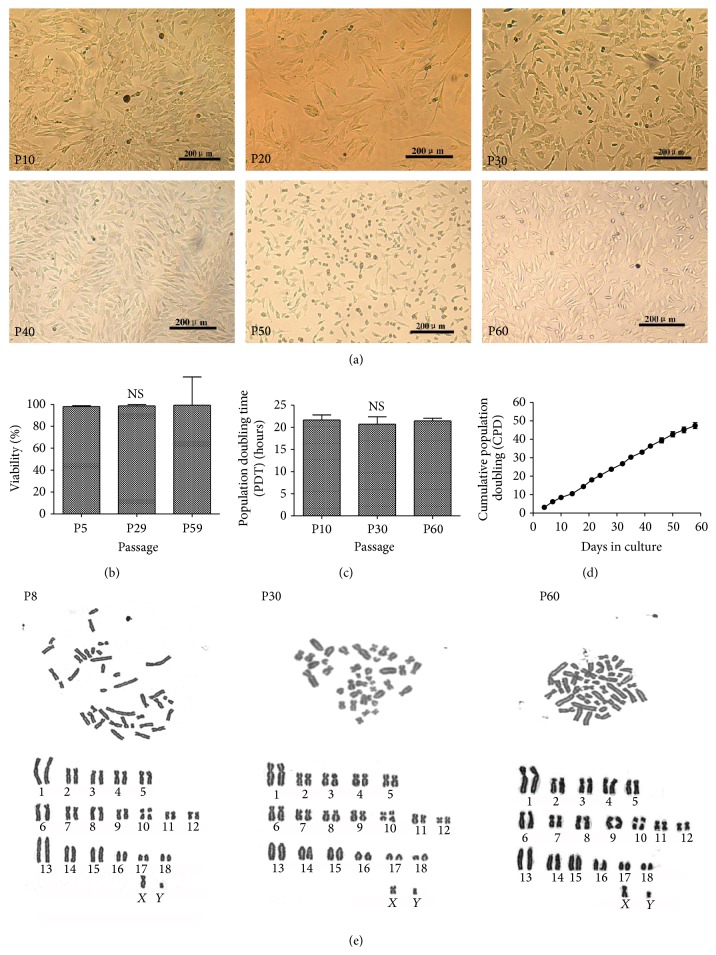
Characterization of porcine DFAT cells during the long-term culture. (a) Different passages of DFAT cells at P10, P20, P30, P40, P50, and P60 uniformly displayed fibroblast-like morphology, bar, 50 *μ*m. (b) Quantification of viability of P5, P29, and P59 DFAT cells by trypan blue staining. (c) Population doubling time (PDT) of DFAT cells at P10, P30, and P60 was 21.68 ± 1.12 h, 20.72 ± 1.69 h, and 21.46 ± 0.63 h, respectively. (d) Cumulative population doubling (CPD) detected from P1 to P16 reached 47.40 ± 1.64 in 58-day culture. (e) The karyotype analysis of P8, P30, and P60 DFAT cells with Giemsa staining. Values are the mean ± SD of triplicate dishes. NS means no significant difference (*p* ≥ 0.05).

DFAT cells were routinely cultured for up to 60 passages* in vitro* and maintained typical spindle-shaped morphology (Figure 3(a)). DFAT cells were fibroblast-like in shape and the cytoplasm was homologous without vacuoles and smudges and displayed a good growth status during the long-term culture.

To examine the cell activity during prolonged culture, cell viability of early (P1–P10), middle (P20–P30), and late (P50–P60) passages was examined by trypan blue staining. Different passages of DFAT cells all remained high viability (>97%) and showed no significance in each other (98.00% ± 0.82% in P5, 98.60% ± 1.14% in P29, and 99.25% ± 0.96% in P59) (*p* > 0.05) (Figure 3(b)), suggesting that DFAT cells maintain high and stable activity during long-term culture* in vitro*.

### 3.4. Growth Kinetics of DFAT Cells during Prolonged Culture* In Vitro*

The population doubling time (PDT) of early passage (P10) was 21.68 ± 1.12 h, which was similar to those of middle (P30) (20.72 ± 1.69 h) and late (P60) passages (21.46 ± 0.63 h) (*p* > 0.05) (Figure 3(c)). Moreover, the cumulative population doubling (CPD) was 47.40 ± 1.64 in 58 days during 16 passages of culture and showed a linear correlation with passage number, indicating a relatively constant population doubling rate over the range studied (*y* = 3.0442*x* − 0.4881, where *y* was the CPD, *x* was passage, and *R* ^2^ = 0.9987) (Figure 3(d)). The results suggest that porcine DFAT cells retain stable growth characteristic and efficient proliferative potential.

### 3.5. Chromosomal Karyotyping Analysis

Chromosomal karyotypes of P8, P30, and P60 DFAT cells were analysed by Giemsa staining. Different passages of porcine DFAT cells all retained a normal diploid karyotype (38 chromosomes) (Figure 3(e)) after long-term culture indicated the stability of genetic characteristic.

### 3.6. Multilineage Differentiation of Porcine DFAT Cells

**Figure 4:**
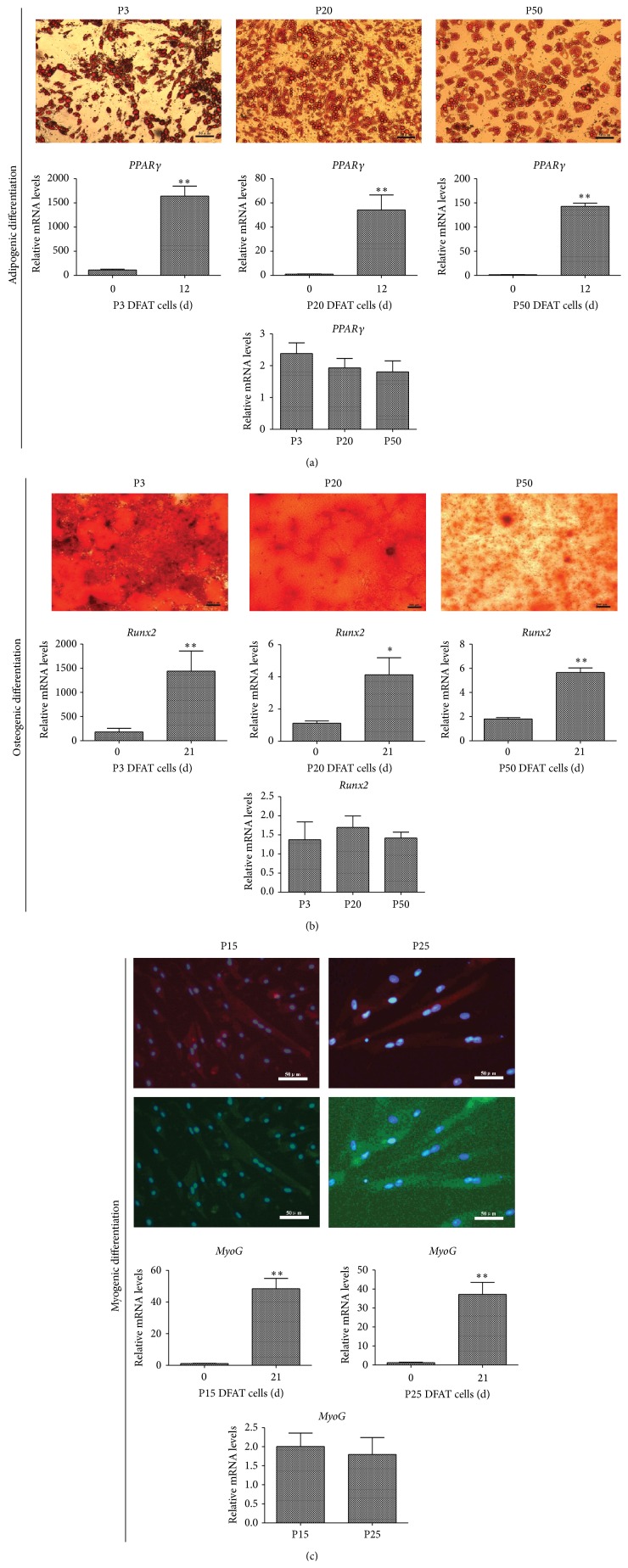
Multilineage differentiation of porcine DFAT cells. (a) Adipogenic differentiation of DFAT cells at P3, P20, and P50 was induced by MDI (high-glucose DMEM containing 10% FBS, 1 *μ*M dexamethasone, 500 *μ*M isobutylmethylxanthine (IBMX), 10 *μ*g/mL insulin, and 200 *μ*M indomethacin) for 12 days. Lipid droplets were stained by Oil Red O, bar, 50 *μ*m. Expression of* PPARγ* mRNA was assessed by real-time PCR. (b) Osteogenic differentiation of DFAT cells at P3, P20, and P50 was induced by induction medium (high-glucose DMEM containing 10% FBS, 0.1 *μ*M dexamethasone, 10 mM *β*-glycerophosphate, and 50 mM ascorbic acid) for 21 days, bar, 200 *μ*m. Expression of* Runx2* mRNA was assessed by real-time PCR. (c) Myogenic differentiation of DFAT cells at P15 and P25 was induced by Galectin-1 for 21 days, expression of myogenic markers was confirmed by immunofluorescence analysis for Desmin and MyHC, and the nuclei were stained by DAPI. Images were merged by Desmin (red) and MyHC (green) with DAPI (blue), bar, 50 *μ*m. Expression of* MyoG* mRNA was assessed by real-time PCR. All values are represented as mean ± SD from three independent experiments. ^∗^ *p* < 0.05, ^∗∗^ *p* < 0.01.

Adipogenic differentiation assessment of P3, P20, and P50 DFAT cells was induced for 12 days. Oil Red O staining represented all passages of DFAT cells accumulated large numbers of lipids and accompanied with significant upregulation of adipogenic differentiation marker* PPARγ* expression after induction. Furthermore, the expression of* PPARγ* showed no differences when inducing adipogenic differentiation for 12 days among the DFAT cells of passages 3, 20, and 50 (Figure 4(a)).

Osteogenic differentiation was performed for P3, P20, and P50 DFAT cells in osteogenic induction medium for 21 days. Mineralized matrix aggregates were observed in all passages of DFAT cells by staining for alizarin red. Furthermore, the expression of osteogenic differentiation marker* Runx2* was significantly increased after induction, and the level of* Runx2* revealed no changes when inducing osteogenic differentiation for 21 days among the DFAT cells of passages 3, 20, and 50 (Figure 4(b)).

Myogenic differentiation of P15 and P25 DFAT cells by Galectin-1 protein for 21 days was induced. Myogenic differentiation was confirmed by the positive staining of Desmin and MyHC, the early and late markers of myogenic differentiation, respectively. Multinucleated cells were observed and this indicated that DFAT cells could be induced into skeletal muscle cells. Real-time PCR data analysis found that the myogenic differentiation marker* MyoG* mRNA was higher after induction 21 day when compared to 0 day. Furthermore, the differentiation ability was changeless among the DFAT cells of passages 3, 20, and 50 (Figure 4(c)).

## 4. Discussion

Adipose tissue has been considered not only an extremely important organ for energy metabolism, but also an endocrine organ that secretes hormones including leptin and adiponectin [16, 17]. Moreover, adipose tissue can be an excellent source for adult stem cells. The DFAT cells, derived from mature adipocytes by ceiling culture* in vitro*, would be a more attractive choice than ASCs [18].

Ceiling culture is commonly used to establish DFAT cells and harvesting high purity of mature adipocytes is crucial [19]. The purity of mature adipocytes could reach 98% confirmed by fluorescence activated cell sorting (FACS) [20]. In this study, we isolated porcine mature adipocytes by collagenase digestion. To eliminate the contaminant cells including preadipocytes and ASCs, multiple cycles of centrifugation and filtration are critical to purify mature adipocytes. Furthermore, differential plating method is effective to obtain mature adipocytes with large single lipid droplet (Figure 1(a)-A). Adipocytes dedifferentiation could be induced by a canonical Wnt ligand, causing the morphology to change into fibroblast-like shape and expression of adipogenic markers to downregulate [21]. Our results turn out that porcine mature adipocytes undergo dedifferentiation during ceiling culture with a series of morphological changes (Figure 1(a)-A–F), causing fibroblast-like porcine DFAT cells to appear. Gene expression changes between mature adipocytes and DFAT cells indicate that DFAT cells lose the functional characteristics of adipocytes, which is consistent with that previously reported [9, 22].

Although DFAT cells are easily established by ceiling culture in many studies and DMEM medium containing 20% FBS was usually used for passaging culture of DFAT cells [9, 11, 12], the optimal culture system for DFAT cells during the long-term subculture* in vitro* has not been established so far. However, high concentration of FBS containing proliferative inhibitors and differentiation promoters may affect the characteristics during the long-term subculture, which resulting in inhibition of cell proliferation, untimely differentiation, and even malignant transformation of MSCs [23, 24]. Basic fibroblast growth factor was recognized as a potent growth factor for cell proliferation and maintained undifferentiated status, which was suitable for cell long-term subculture* in vitro* [25]. In this study, culture mediums of DMEM medium containing 15% FBS and 10 ng/mL bFGF are used for long-term culture of porcine DFAT cells. In this culture condition, porcine DFAT cells can be propagated for 60 passages and still maintain the normal spindle-shaped morphology with high efficient cell viability (>97%).

DFAT cells possess high capacity of proliferation* in vitro* [26]. Growth kinetics of porcine DFAT cells including CPD and PDT usually evaluated the proliferative capacity. The PDT at all passages of DFAT cells is less than 24 h and is similar to the continuous cell line, which is much smaller than human DFAT cells (30–70 h) and ASCs (40–120 h) [8, 9]. In addition, the CPD of porcine DFAT (47.40 ± 1.64 in 58 days after 16 passages) is much higher than the one reported about human DFAT cells (14.5 ± 7.3 in 88 days after 5 passages) [11]. These above mentioned results reveal that porcine DFAT exhibit potent proliferative potential even after long-term culture* in vitro*.

Previous studies found that DFAT cells that possess properties similar to MSCs have been multilineage potential, including adipocytes, osteoblasts, chondrocytes [9, 11, 27], myocytes [28], smooth muscle cells [29], cardiomyocytes [12], and vascular endothelial cells [30]. Moreover, due to the potent adipogenic differentiation capacity, porcine DFAT cells were used as preadipocytes models for adipogenic differentiation [26]. However, to our knowledge, apart from adipogenic differentiation, different lineages differentiation of DFAT cells has not been reported in pigs. Therefore, we evaluate adipogenic, osteogenic, and myogenic differentiation of porcine DFAT cells during long-term culture* in vitro* (Figure 4). The results indicate that porcine DFAT cells maintain ability to undergo differentiation of adipocytes, osteoblasts, and myocytes by prolonged passaging. In particular, the visible mineralized matrix aggregates and multinucleated myotubes were for the first time to confirm the osteogenic and myogenic differentiation of DFAT cells in pigs.

In summary, we success to establish the porcine DFAT cells by ceiling culture and provide evidence of morphology, cell viability, growth kinetics, differentiation, and karyotype, revealing that porcine DFAT cells exhibit efficient proliferative activity, multipotent differentiation to adipocytes, osteoblasts, and myocytes, normal diploid karyotype during the long-term culture* in vitro*. Because of the abundance and the easy accessibility of mature adipocytes, porcine DFAT cells can be an excellent stem cell model applied to tissue engineering and regenerative medicine.
